# Supplementary material for: Addressing Vitamin B12 deficiency through aeroponic fortification of a salad crop (Pisum sativum)
Source: Commun Biol. 2026 Mar 6;9:544. doi: 10.1038/s42003-026-09764-y (PMC13096416; doi:10.1038/s42003-026-09764-y)
Supplement: Supplementary file 2 — Supplementary Information [file 42003_2026_9764_MOESM2_ESM.pdf]

Supplementary Information for "Addressing Vitamin B_{12} deficiency through aeroponic fortification of a salad crop (*Pisum sativum*)."

Bethany M. Eldridge, Sree Gowrindah Javaddi, Natalia Perez-Moral, Jessie Sweetman, Luíza Lane de Barros Dantas, Shikha Saha, Deirdre A. Lynch, Thomas Hunt, Sophie E. Clough, Jemal Toussaint, Andy Worrall, Lillian R. Manzoni, Nigel Robinson, Keara A. Franklin, Cathrina Edwards, Jonathan Clarke, Jack Farmer, Martin Warren, Antony N. Dodd

Contents

Supplementary Figures 1-3
Supplementary Tables 1 and 2

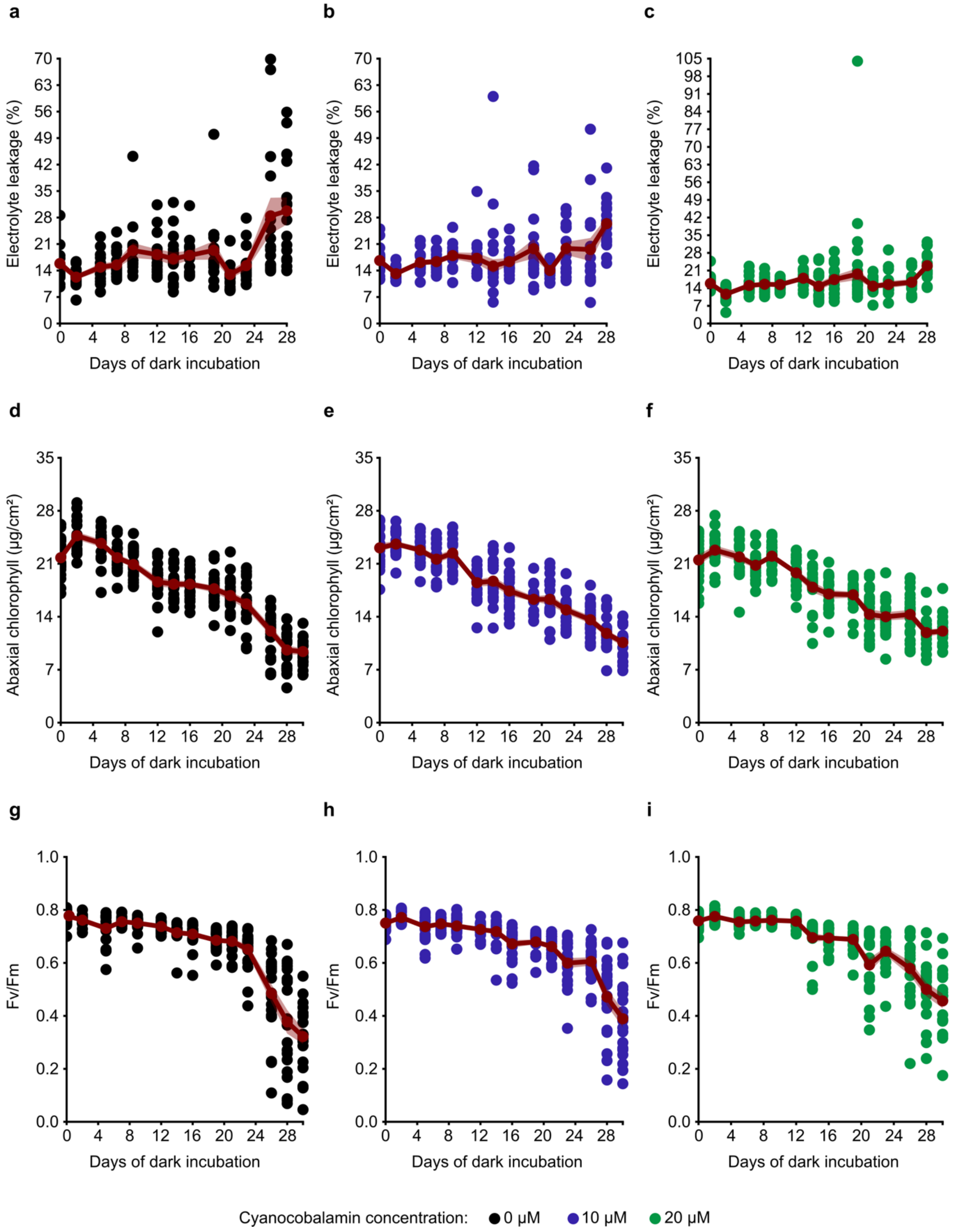

Supplementary Figure 1. Senescence measures during simulated cold-chain storage, showing individual data points. These data underlie Figure 4c-e. (a-c) Proportion of total electrolytes released from pea shoots supplemented with several concentrations of cyanocobalamin, compared with no treatment control, during 28 days of simulated cold chain storage. Measurements on successive days were from separate plants, to avoid resampling the same plant material. (d-f) Mean abaxial chlorophyll content of pea shoots supplemented with several concentrations of cyanocobalamin, compared with no treatment control, during 28 days of simulated cold chain storage. Chlorophyll content was estimated using an optical

method (Dualex instrument). (g-i) Maximum efficiency of photosystem II (PSII) (Fv/Fm) of pea shoots, in plants supplemented with several concentrations of cyanocobalamin, compared with no treatment control, across 28 days of simulated cold chain storage. Red circles indicate mean values, and associated red shading indicates s.e.m.

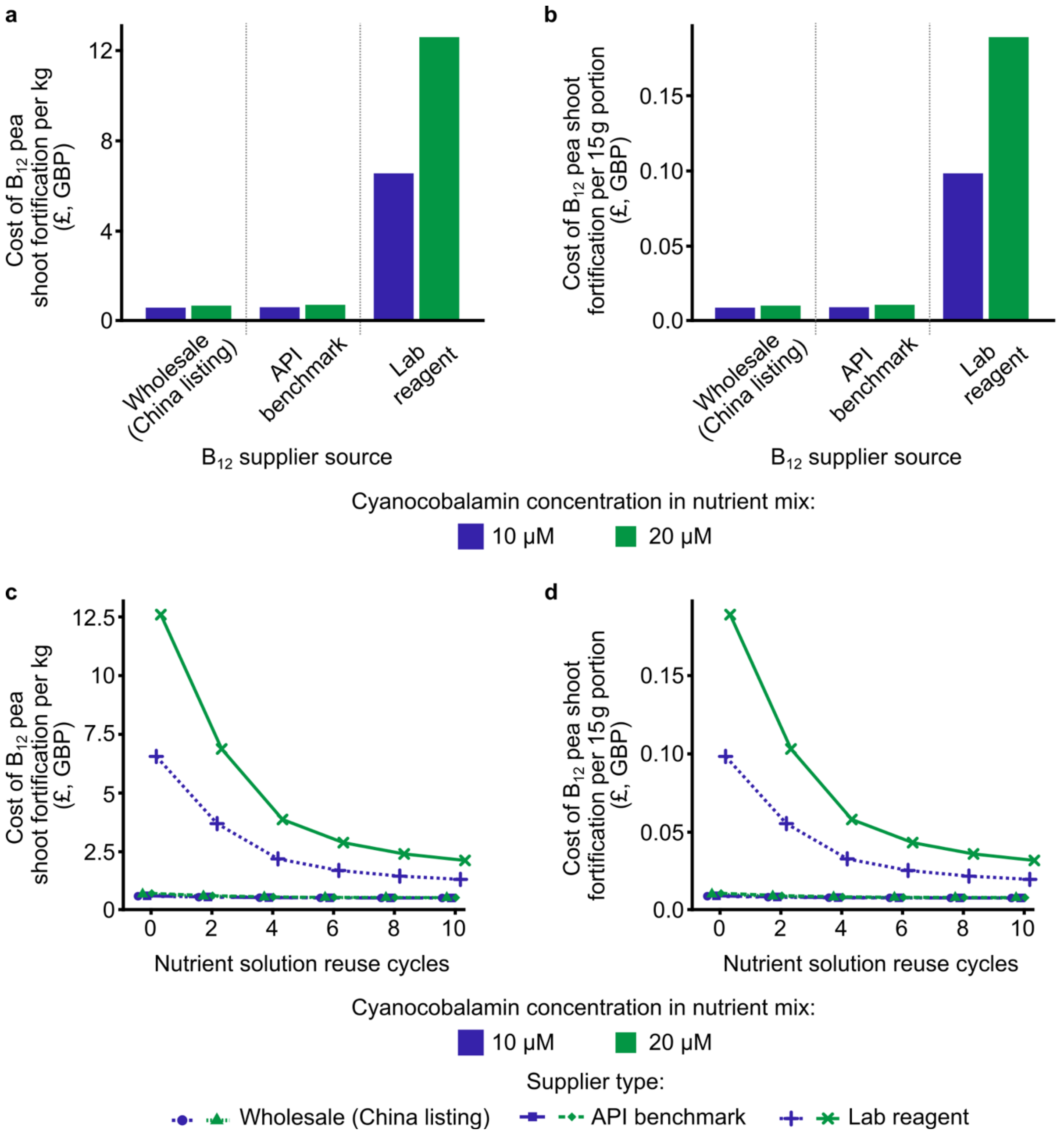

Supplementary Figure 2. Techno-economic assessment of Vitamin B_{12} fortification of pea shoots using aeroponics. (a) Fortification cost per kilogram of pea shoots (10 µM and 20 µM cyanocobalamin treatments) with no nutrient recycling, across three cyanocobalamin price tiers. (b) Fortification cost per 15 g portion of pea shoots with no nutrient recycling, across the same cyanocobalamin price tiers. (c) Total additional cost per kilogram of pea shoots (10 µM and 20 µM cyanocobalamin treatments) as a function of reuse cycles (2–10), across three cyanocobalamin price tiers, compared with no solution reuse. (d) Total additional cost per 15 g portion of pea shoots (10 µM and 20 µM treatments) as a function of nutrient solution reuse cycles (0 - 10). Reuse scenarios incorporate assumed cumulative B_{12} losses of 5–25%. (c, d) Graph lines represent proportional cost savings for 10 µM and 20 µM treatments across cyanocobalamin price tiers. Costs are in £, GBP.

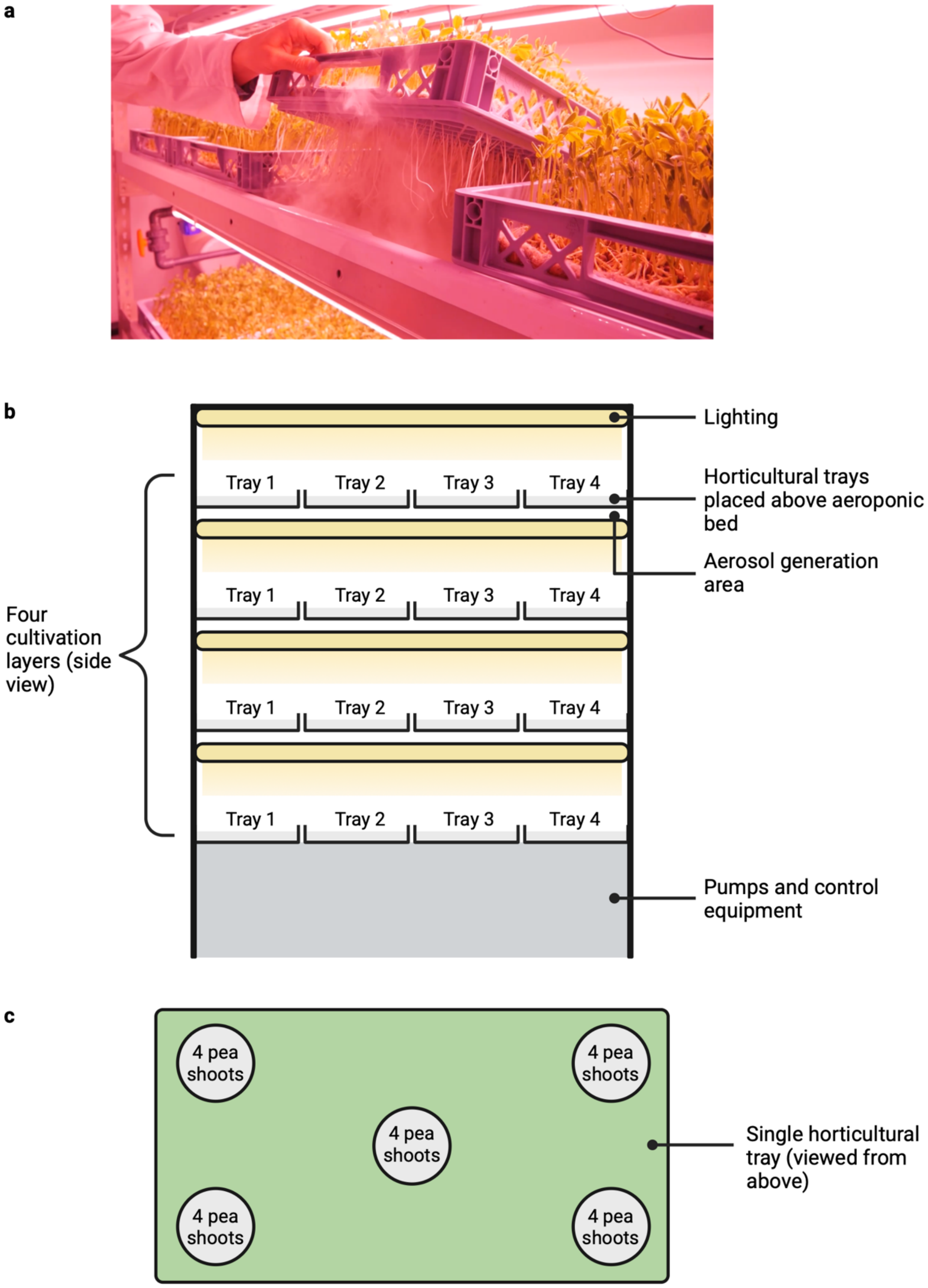

Supplementary Figure 3. Aeroponic cultivation, stack configuration, and sampling approach. (a) Photograph of aeroponic cultivation system with pea shoots in growing trays. (b) Design of aeroponic stacks used for experimental work. The system comprised four layers, each of which carried four separate horticultural trays, with lighting suspended above each layer. (c) Sampling strategy employed within study to obtain information about B_{12} content and plant performance from across each horticultural tray. Created in BioRender. Dodd, A. (2026) https://BioRender.com/s75k842.

**Crop species**	**B_{12} supplementation method**	**B_{12} supply solution concentration (µM)**	**B_{12} content in crop (per g fresh weight)**	**Reference**
Glycine max (soybean leaves)	Hydroponic cultivation for 24 h	10 µM	9.8 µM	Mozafar and Oertli (1992) [24]
Raphanus sativus (daikon radish sprouts)	Seed soaking for up to 6 hours	18.40 µM<br>147.60 µM	0.20 µM<br>1.30 µM	Sato, Kudo and Muramatsu (2004) [23]
Lactuca sativa (lettuce leaves)	Hydroponic cultivation for 24 h	1.00 µM<br>5.00 µM<br>10.00 µM	38.60 µM<br>164.60 µM<br>154.90 µM	Bito et al. (2013) [25]
Spinacia oleracea<br>(spinach leaves)	Hydroponic cultivation for 1-2 days	9.80 µM	approx. 3.8 µM	Zheng et al. (2021) [26]
Pisum sativum (pea shoots)	Aeroponic cultivation	10.00 µM	0.53 µM	Eldridge et al. (this study)
Pisum sativum (pea shoots)	Hydroponic cultivation	10.00 µM	0.47 µM	Eldridge et al. (this study)
Raphanus sativus (daikon radish sprouts)	Seed soaking for 6 hours	147.6 µM	0.50 µM	Patent EP1371283 [28]
Broad spectrum: azuki beans, amaranth, lucerne, picris, watercress, buckwheat, various peas, fenugreek, soy, barley, oat, millet, pumpkin, chickpea, cabbage, lentils, linseed, maize, rice, radish, rye, sesame, mustard, sunflower, wheat, quinoa	Soaking seed in B12 containing solution for 1-48 h			Patent US8679555 [27]

Supplementary Table 1. Comparison of various published approaches for crop fortification with vitamin B12. Table extended from [25].

Nutrient	Hydromax nutrient A+B mix (%, w/v)
Total nitrogen (N)	4.9 %
Nitrate nitrogen (NH_4)	4.3 %
Ammoniacal nitrogen (NO_3)	0.6 %
Phosphorous pentoxide (water soluble, P_2O_5)	2.3 %
Potassium oxide (K_2O)	6.8 %
Calcium oxide (CaO)	3.3 %
Copper (Cu) chelated by EDTA	-
Iron (Fe) chelated by EDTA	-
Iron (Fe) chelate by EDDHA	0.067 %
Manganese (Mn) chelated by EDTA	0.023 %
Molybdenum (Mo) chelated by EDTA	0.001 %
Zinc (Zn) chelated by EDTA	0.008 %
Magnesium oxide (Mgo)	1.1 %
Sulfur Trioxide (SO_3)	2.8 %
Boron (B)	0.008 %
Cobalt (Co)	0.001 %
Nickel (Ni)	0.001 %

Supplementary Table 2. Composition of commercially-available plant growth nutrient mix used for this study.
