# Supplementary material for: Addressing Vitamin B12 deficiency through aeroponic fortification of a salad crop (Pisum sativum)
Source: Commun Biol. 2026 Mar 6;9:544. doi: 10.1038/s42003-026-09764-y (PMC13096416; doi:10.1038/s42003-026-09764-y)
Supplement: Supplementary file 3 — Description of Additional Supplementary Materials [file 42003_2026_9764_MOESM3_ESM.pdf]

Description of Additional Supplementary Files

File name: Supplementary Data 1

Description: All source data for this study.
